# Supplementary material for: A Genome-Scale Metabolic Model of Methanoperedens nitroreducens: Assessing Bioenergetics and Thermodynamic Feasibility
Source: Metabolites. 2022 Mar 31;12(4):314. doi: 10.3390/metabo12040314 (PMC9024614; doi:10.3390/metabo12040314)
Supplement: Supplementary file 1 [file metabolites-12-00314-s001.zip › Supplementary Table S1-S2.pdf]

Table 1. Oxidation/reduction midpoint potentials for the reactions in reverse methanogenesis pathway.

Oxidant	Reductant	Em(mv)
CoM-S-S-CoB	CoM-SH + CoB-SH	-140
F420	$F420H_2$	-380
Fd_{ox}	Fd_{red}	-520
MQ	MQH_2	-80
NO_3^-	NO_2^-	+420

Table 2. Gibbs free energy of the reactions in reverse methanogenesis pathway in *M. nitroreducens.* The Gibbs free energy of Mcr, Mtr and Mer + Mtd + Mch, Ftr + Fmd are published values. HdrDE, HdrABC, NarGH and Fqo are calculated with the published standard midpoint potentials presented in Supplementary Table 1, using ΔG = -n $F\Delta E$. F is the Faraday constant, 0.09648kJ/eV, n is the number of moles of electrons transferred in the reaction.

Enzyme	Reaction	ΔG°′ (kJ/mol)
Mcr	CH_4[c] + CoM-S-S-CoB[c] <=> CoB-SH[c] + CH_3-CoM[c]	+45
Mtr	CH_3-CoM[c] + H_4MPT[c] + 2 Na^+[e] <=> CH_3-H_4MPT[c] + CoM-SH[c] + 2 Na^+[c]	+30
HdrDE	CoB-SH[c] + CoM-SH[c] + 2 H^+[e] + MQ[c] <=> CoM-S-S-CoB[c] + MQH_2[c] + 2 H^+[c]	-11.58
HdrABC	CoB-SH[c] + CoM-SH[c] + 2 Fd_{red}[c] + 2 F420[c] <=> CoM-S-S-CoB[c] + 2 Fd_{ox}[c] + 2 $F420H_2$[c]	+38.59
NarGH	NO_3^-[e] + MQH_2[c] + 4 H^+[c] <=> NO_2^-[e] + MQ[c] + H_2O[c] + 4 H^+[e]	-96.48
Mer+Mtd +Mch	CH_3-H_4MPT[c] + 2 F420[c] <=> CHO-H_4MPT[c] + 2 $F420H_2$[c]	-5.3
Ftr+Fmd	CHO-H_4MPT[c] + Fd_{ox}[c] + H_2O[c] <=> CO_2[c] + H_4MPT[c] + Fd_{red}[c]	-32
Fqo	$F420H_2$[c] + MQ[c] + 3 H^+[c] <=> F420[c] + MQH_2[c] + 3 H^+[e]	-57.87
