# Supplementary figures and images for: A Genome-Scale Metabolic Model of Methanoperedens nitroreducens: Assessing Bioenergetics and Thermodynamic Feasibility
Source: Metabolites. 2022 Mar 31;12(4):314. doi: 10.3390/metabo12040314 (PMC9024614; doi:10.3390/metabo12040314)

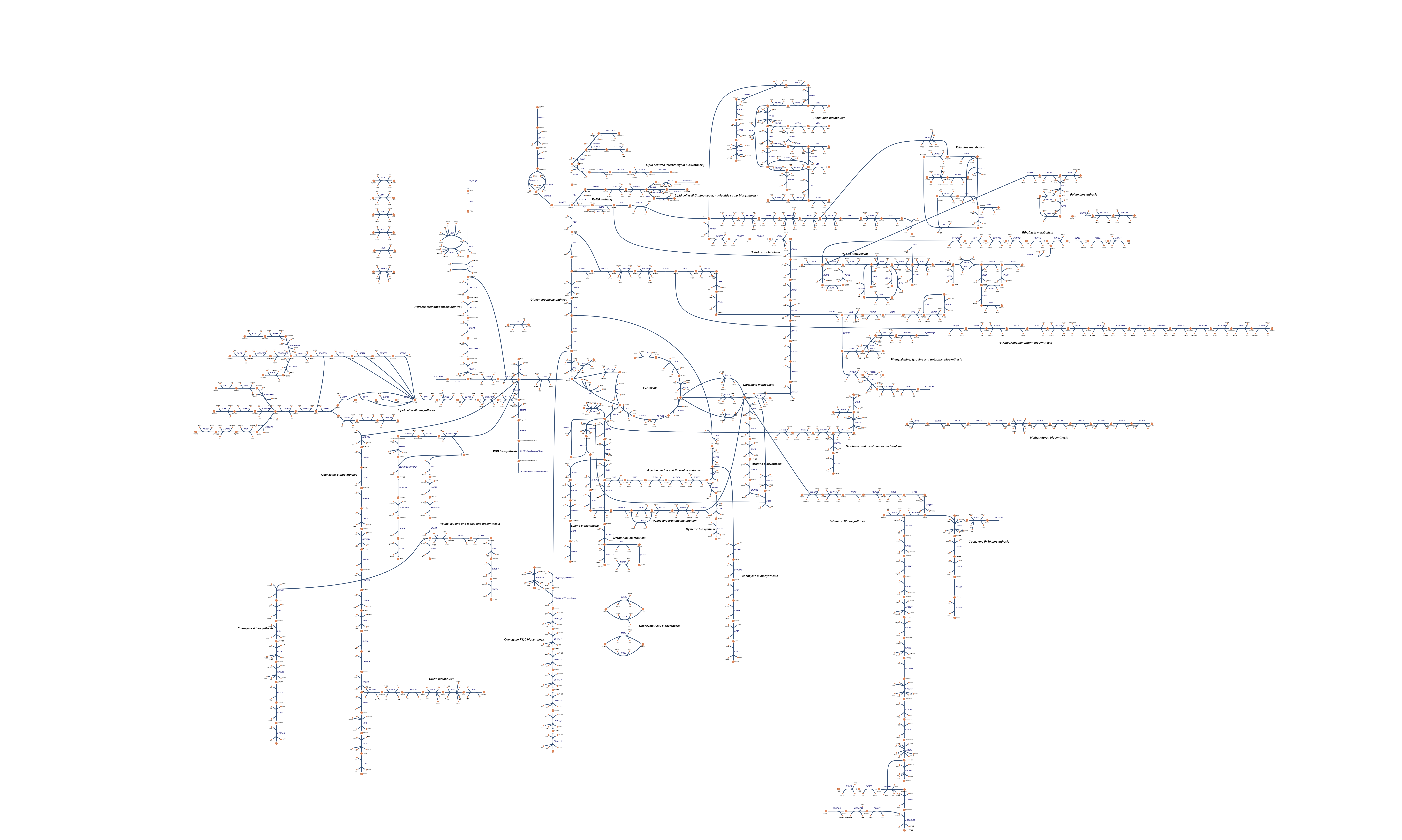

Supplement: Supplementary file 1 [file metabolites-12-00314-s001.zip › Supplementary Figure S1 GEM network map.png]
